# Evaluation of Suitable Reference Genes for Quantitative Real-Time PCR in Various Tissues of *Apocynum venetum*

**DOI:** 10.3390/genes15020231

**Published:** 2024-02-11

**Authors:** Xiaoshuang Li, Tingting Zhang, Li Jiang, Guizhi Fan

**Affiliations:** 1 Key Laboratory of Saline-Alkali Vegetation Ecology Restoration, Ministry of Education, Northeast Forestry University, Harbin 150040, China1010845451@126.com (T.Z.); 2 State Key Laboratory of Desert and Oasis Ecology, Xinjiang Institute of Ecology and Geography, Chinese Academy of Sciences, Urumqi 830011, China

**Keywords:** *Apocynum venetum*, gene expression, real-time PCR, reference genes, validation

## Abstract

*Apocynum venetum* L. is an economically valuable plant with tolerance to drought and salinity. Its leaves are utilized in tea production and pharmaceuticals, while the stem bark serves as a high-quality fiber material. To gain insights into the gene expression patterns of *A. venetum* using quantitative real-time PCR (qRT-PCR), it is crucial to identify appropriate reference genes. This study selected nine candidate genes, including α-tubulin (*TUA*), β-tubulin (*TUB*), actin (*ACT*), cyclophilin (*CYP*), elongation factor-1α (*EF-1α*), the B family of regulatory subunits of protein phosphatase (*PPP2R2*, *PPP2R3*, and *PPP2R5*), and phosphoglycerate kinase (*PGK*), to determine the most appropriate reference genes in the leaf, stem, and root tissues of *A. venetum*. A comprehensive ranking by geNorm, NormFinder, BestKeeper, and RefFinder software and Venn diagrams was used to screen more stable reference genes in different tissues. The two most stable reference genes were *CYP* and *TUA* in leaves, *PGK* and *PPP2R3* in stems, and *TUA* and *EF-1α* in roots, respectively. The relative expression values of the four genes involved in proline metabolism under polyethylene glycol treatment were used to validate the screened reference genes, and they exhibited highly stable expression levels. These findings represent the first set of stable reference genes for future gene expression studies in *A. venetum*. They significantly contribute to enhancing the accuracy and reliability of gene expression analyses in this economically important plant species.

## 1. Introduction

The exploration of gene expression patterns in biology is expected to offer valuable insights into intricate regulatory networks, thereby assisting in the identification of genes that are pertinent to various biological processes [1]. Quantification of specific mRNA is a fundamental requirement for analyzing gene expression. At present, Northern blotting, microarray analysis, quantitative real-time PCR (qRT-PCR), and RNA sequencing are employed to quantify specific mRNA levels in diverse tissues of an organism [2,3,4]. Among them, qRT-PCR emerges as a highly dependable technique for analyzing gene expression, primarily owing to its sensitivity, reproducibility, specificity, and capacity for high yield [5,6]. Additionally, it underscores the importance of data normalization for obtaining reliable gene expression values in qRT-PCR analysis. The preferred method for error control mentioned is normalization to either a solitary internal reference gene or a group of multiple reference genes. This normalization step is crucial to mitigate errors and ensure the accuracy of the gene expression data obtained through qRT-PCR [7,8,9]. For reference genes, no universal gene is suitable for every species [10,11]. Hence, it is crucial to validate appropriate reference genes under diverse conditions to ensure the acquisition of biologically meaningful expression data.

*Apocynum venetum*, which belongs to the Apocynaceae family, is widely distributed across salt-barren zones, desert steppes, and alluvial flats in the Mediterranean region and Northwestern China [12,13,14]. *A. venetum* has the reputation of “king of wild fiber” and has been extensively utilized in traditional Chinese and Uygur medicine because of its anti-inflammation, antioxidative, antihypertensive, anticancer, antiviral, and bactericidal effects [15,16,17]. Despite its medicinal significance, limited attention has been given to the exploration of *A. venetum* genes and their expression patterns to unveil their biological functions [14]. Identifying stable candidate reference genes is essential for uncovering the pivotal genes linked to stress resistance or the biosynthesis of active components in *A. venetum* at the gene expression level. However, there is a deficiency in research focused on the screening of candidate reference genes for this purpose. 

In this study, nine reported genes, α-tubulin (*TUA*), β-tubulin (*TUB*), actin (*ACT*), cyclophilin (*CYP*), elongation factor-1α (*EF-1α*), B family of regulatory subunits of the protein phosphatase (*PPP2R2*, *PPP2R3*, and *PPP2R5*), and phosphoglycerate kinase (*PGK*), were used to identify the most stable reference genes for the normalizing of gene expression data in different tissues (leaf, stem, and root) of *A. venetum.* The stability ranking of potential reference genes was conducted using geNorm, NormFinder, BestKeeper, and RefFinder software. This research will establish a strong empirical basis for future investigations into gene expression in *A. venetum* through the utilization of qRT-PCR.

## 2. Materials and Methods

### 2.1. Plant Materials and PEG6000 Treatment

In vitro-cultured plantlets of *A. venetum* were cultured on woody plant medium (WPM) supplemented with a sucrose concentration of 20 g L^−1^. The plantlet culture was nurtured from germinated seeds collected in Xinjiang, China, in 2017. Prior to autoclaving, the pH of the medium was adjusted to 5.8 ± 0.1. All explants were cultured under controlled conditions at a temperature range of 25 ± 2 °C and subjected to a light/dark cycle of 16 h/8 h. 

Plantlets that were approximately 15 cm in height were selected to treatment by 20% polyethylene glycol (PEG) 6000 for 24 h. Leaves, stems, and roots were individually harvested and rapidly frozen using liquid nitrogen for RNA extraction.

### 2.2. Total RNA Extraction and cDNA Synthesis

The CTAB method was employed to extract the total RNA from the samples [18], followed by treatment with RNase-free DNase I (Takara, San Jose, CA, USA) as per the manufacturer’s instructions to eliminate genomic DNA. The concentration and quality of the RNA samples were determined using a DS-11 spectrophotometer (Denovix, Wilmington, DE, USA), assessing both their concentration and 260/280 ratio. Furthermore, confirmation of RNA sample integrity was achieved through agarose gel electrophoresis using a 1.5% (*w*/*v*) gel. For cDNA synthesis, we utilized the PrimeScript™ RT reagent kit from Takara Bio Inc., Shiga, Japan, following the manufacturer’s guidelines.

### 2.3. qRT-PCR Primer Design and Analysis

Six traditional (*TUA*, *TUB*, *ACT*, *CYP*, *EF-1α*, and *PGK*) and three potential (*PPP2R2*, *PPP2R3*, and *PPP2R5*) reference genes were chosen based on the full-length transcriptome data of *A. venetum* (PRJNA650225). To validate the transcriptome data, we cloned five of the above genes, namely *CYP* (MW012394), *TUA* (MW012395), *PGK* (MW012392), *PPP2R3* (MW012393), and *AvEF-1α* (MW187080), whose sequences were consistent with the transcriptome-sequencing data.

The primer pairs were designed according to specific criteria to ensure optimal performance. These criteria included amplicon lengths ranging from 100 bp to 300 bp, guanine–cytosine contents between 40% and 60%, primer lengths spanning from 20 bp to 22 bp, a temperature difference of less than 1 °C for each pair, and a melting temperature (Tm) within the recommended range of 59–62 °C as suggested by previous studies [7,19]. Detailed information about the primer pairs developed in this study can be found in Table 1. The qRT-PCR reactions were performed using the Applied Biosystems 7500 RT-PCR System (Thermo Fisher, Waltham, MA, USA). PCR amplification followed standard cycling conditions, which involved an initial denaturation step at 95 °C for 30 s, followed by 40 cycles consisting of denaturation at 95 °C for 5 s and annealing/extension at 60 °C for 34 s. 

### 2.4. Stability Analysis

The expression levels of the candidate reference genes in various tissues were determined by analyzing the cycle threshold values (Ct). The stability analysis of these nine candidate reference genes was conducted using geNorm (version 3.5), NormFinder (version 20), and BestKeeper (version 1). Additionally, we utilized the RefFinder web tool (https://www.heartcure.com.au/reffinder/) (accessed on 15 March 2021), which integrated geNorm, NormFinder, BestKeeper, and the comparative ΔCt method algorithms, to comprehensively evaluate the qRT-PCR data and generate a ranking of transcript stability [20]. A smaller ranking value indicated higher stability for a given reference gene. Furthermore, we employed a Venn diagram approach (http://bioinformatics.psb.ugent.be/webtools/Venn/) (accessed on 15 March 2021) to summarize the consensus among all algorithms in selecting the best reference genes. 

The upregulation of key enzyme genes involved in plant proline metabolism was observed during drought conditions [21]. Therefore, four key enzyme genes of proline metabolism, namely, Δ^1^-pyrroline-5-carboxylate synthase (P5CS), ornithine δ-aminotransferase (OAT), proline hydroxylase (PHD), and glutamic acid decarboxylase (GAD), were selected to assess the stability of potential reference genes under 20% PEG 6000 treatment. The primer details for these genes are provided in Table 1. Gene expression data were calculated with the 2^−ΔΔCt^ method [22].

### 2.5. Statistical Analysis

All experiments were conducted with 3 biological replicates and 3 technical replicates. The figures present the average values ± standard error. Tukey’s tests were employed for conducting multiple comparisons among means. Statistical significance was determined at a threshold of *p* < 0.05.

## 3. Results

### 3.1. Selection of Candidate Reference Genes of A. venetum

**Figure 1:**
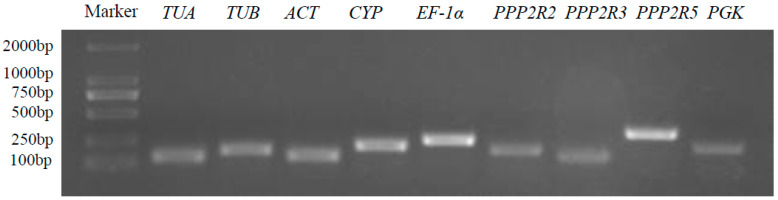
PCR amplification outcomes of 9 potential reference genes from *A. venetum*. The integrity of all RNA samples was verified using agarose gel electrophoresis with a concentration of 1.5% (*w*/*v*) (Figure S1).

Nine potential reference genes (*TUA*, *TUB*, *ACT*, *CYP*, *EF-1α*, *PPP2R2*, *PPP2R3*, *PPP2R5*, and *PGK*) were selected from *A. venetum* transcriptome datasets according to the criteria of published papers. The details of these candidate reference genes can be found in Table 1. The qRT-PCR products of candidate reference genes ranged from 135 bp (*ACT*) to 279 bp (*PPP2R5*), and their electrophoresis bands were bright and clear without primer dimers or impurities (Figure 1). Furthermore, the specificity of the primer pairs was further confirmed by melt curve analysis, which showed a single peak for each gene amplification product (Figure S2). The confirmed specificity of the primers for all nine candidate reference genes solidified their suitability and reliability for qRT-PCR applications.

### 3.2. Expression Levels of Candidate Reference Genes

**Figure 2:**
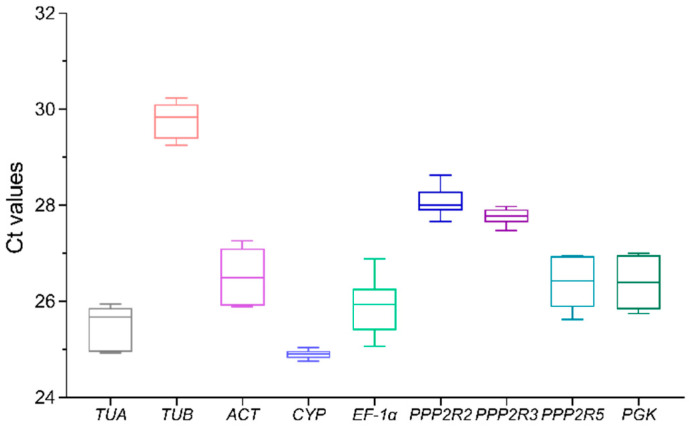
Ct values for nine potential reference genes in all plantlet samples of *A. venetum*. The bigger the box, the greater the variation. The maximum and minimum Ct values are represented by whiskers, while the median values are indicated by a line across the box.

The assessment of expression levels of the candidate reference genes in *A. venetum* plantlets’ leaves, stems, and roots was conducted using cycle threshold (Ct) values. These Ct values represent the number of cycles required for fluorescence to reach a predetermined detection threshold. Figure 2 displays the Ct data from three replicated reactions with nine primer pairs. The mean Ct values ranged from 24.90 (CYP) to 29.78 (TUB). It is worth noting that among the nine candidate reference genes, CYP demonstrated the highest level of gene expression.

### 3.3. Stability Analysis of Candidate Reference Genes 

**Figure 3:**
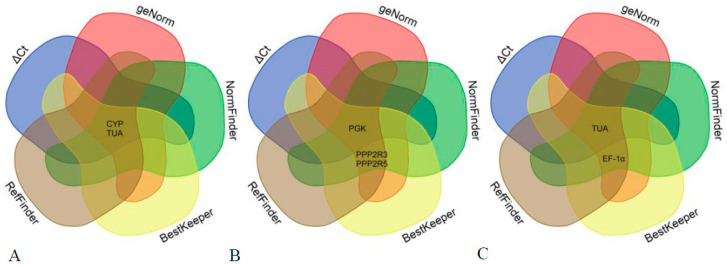
Candidate reference genes in various tissues of *A. venetum* using a Venn diagram. (**A**) Venn diagram of candidate reference genes in leaves of *A. venetum*; (**B**) Venn diagram of candidate reference genes in stems of *A. venetum*; (**C**) Venn diagram of candidate reference genes in roots of *A. venetum*.

The gene expression stability of nine potential reference genes was extensively evaluated and ranked using geNorm, NormFinder, and BestKeeper software (Figures S3–S5). The ultimate ranking was determined through the utilization of RefFinder software and a Venn diagram generated from the aforementioned programs (Table 2 and Figure 3). 

Lower values measured in geNorm, NormFinder, and BestKeeper software represented higher expression stability in reference genes. As shown in Table 2, the selection of the most stable reference genes differed among various plant tissues: *PGK* and *CYP* in leaves, *PGK* and *PPP2R3* in stems, and *EF-1α* and *TUA* in roots. The two most stable reference genes varied across different plant tissues: *PPP2R5* and *CYP* in leaves, *PPP2R3* and *PGK* in stems, *CYP* and *TUA* in roots. The most two stable reference genes were *CYP* and *PGK* in leaves, *PPP2R5* and *PPP2R3* in stems, *ACT* and *TUA* in roots.

The selection of the two most reliable reference genes differed slightly among the three algorithms mentioned above. Therefore, it was crucial to obtain a comprehensive ranking. The results obtained from RefFinder software and Venn diagram analysis using all algorithms were nearly identical. In leaf samples, *CYP* and *TUA* were identified as the two most stable reference genes, while in stem samples, *PGK* and *PPP2R3* showed high stability. In root samples, *TUA* and *EF-1α* were found to be the most stable reference genes.

The M values were obtained from geNorm, the SV values were determined by NormFinder, SD represented standard deviation in BestKeeper, CV indicated the coefficient of variation in BestKeeper, and the ultimate comprehensive ranking was computed using RefFinder.

### 3.4. Determination of the Optimal Number of Reference Genes for Normalization

**Figure 4:**
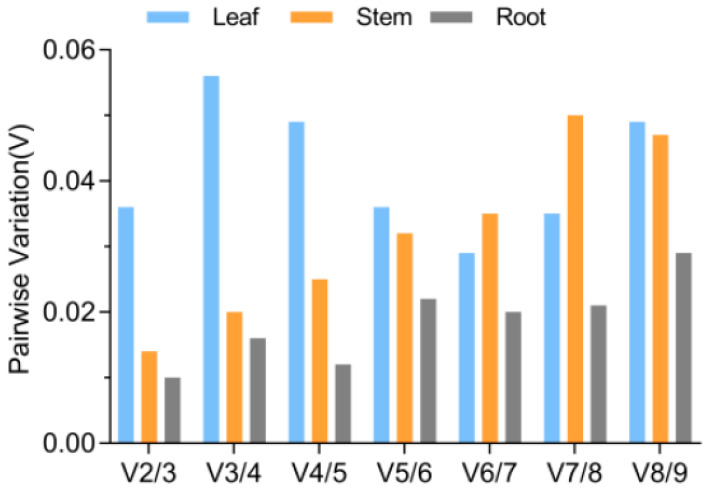
Assessment of potential reference genes through pairwise comparison calculated by the geNorm software. The analysis involved comparing the normalization factors NFn and NFn + 1 in all tested conditions to determine their pairwise variation (Vn/Vn + 1).

The optimal number of reference genes was determined using the geNorm software through a pairwise comparison (Vn/n + 1). According to Gong et al. (2016) [23], if the threshold value is lower than 0.15, there is no need for additional reference genes for normalization. Figure 4 illustrates that the V-values in leaves, stems, and roots were all below 0.15. Hence, two reference genes were sufficient to normalize qRT-PCR data across all analyzed tissues.

### 3.5. Validation of the Stability of the Chosen Reference Genes

**Figure 5:**
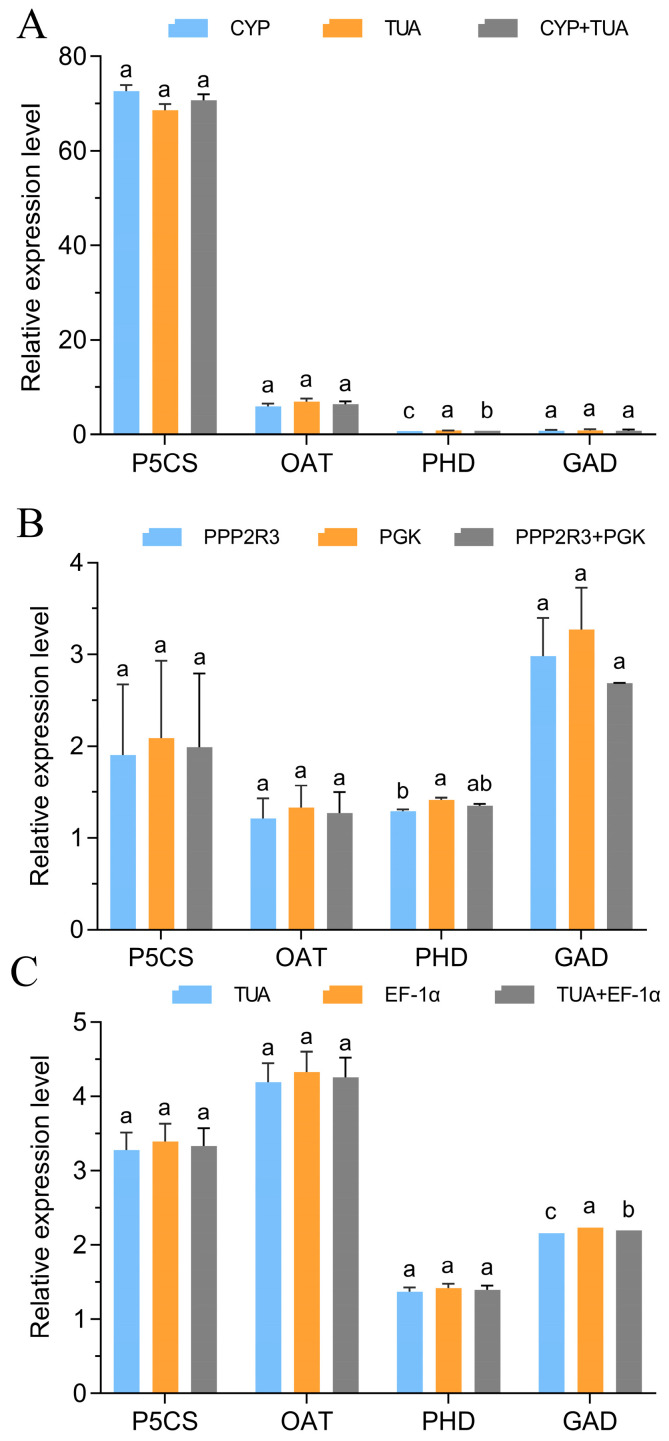
Relative expression levels of *P5CS*, *OAT*, *PHD*, and *GAD*. (**A**) Expression level of *P5CS*, *OAT*, *PHD*, and *GAD* normalized by *CYP* and *TUA* in leaves. (**B**) Expression level of *P5CS*, *OAT*, *PHD*, and *GAD* normalized by *PGK* and *PPP2R3* in stems. (**C**) Expression level of *P5CS*, *OAT*, *PHD*, and *GAD* normalized by *TUA* and *EF-1α* in roots. The error bars represent the mean of three biological replicates ± SD. Different letters show significant differences among gene expression levels normalized by two selected reference genes, individually and jointly (*p* < 0.05, Tukey’s test).

The gene expression levels of four genes (*P5CS*, *OAT*, *PHD*, and *GAD*) associated with proline metabolism were investigated across various tissues subjected to 20% PEG treatment for 24 h. This analysis aimed to validate the previously selected reference genes. As shown in Figure 5, except for *PHD* in leaves and stems and *GAD* in roots, the gene expression normalized by two selected reference genes had no remarkable differences individually and jointly. For example, the gene expression of *P5CS* normalized by *CYP* or *TUA* in leaves under 20% PEG treatment was almost similar. The results also exhibited higher expression stability of selected normalizing reference genes in their corresponding tissues. Additionally, gene expression of *P5CS* was higher than that of the other three genes in leaves or whole plantlets under 20% PEG treatment for 24 h.

## 4. Discussion

The genomic and transcriptomic information for *A. venetum* has been extensively documented [24,25]. qRT-PCR is widely recognized as a powerful technique that enables the accurate quantification of gene expression, providing more precise expression profiles of genes of interest. To minimize biological interferences and remove variation, it is essential to employ reference genes as internal controls to normalize mRNA levels during qRT-PCR analysis [7,8,26]. However, the suitability of a single reference gene may vary across different plant species, tissues, and environmental conditions [10,11]. Therefore, it is crucial to validate candidate reference genes for their stability before implementing them in qRT-PCR experiments.

*TUA*, *TUB*, *ACT*, *CYP*, *EF-1α*, *PPP2R2*, *PPP2R3*, *PPP2R5*, and *PGK* have been validated as stable reference genes in plants [8,9,26,27]. Based on a lack of qRT-PCR reference genes in *A. venetum*, the above nine candidate genes were screened and identified by stability analysis in order to determine the optimal reference genes in the leaves, stems, and roots of *A. venetum.* All nine genes exhibited varying levels of expression across different tissues in *A. venetum*. Suitable reference genes in different tissues were found using a comprehensive ranking by geNorm, NormFinder, BestKeeper, and RefFinder software and Venn diagrams. The two most stable reference genes in leaves, stems, and roots were *CYP* and *TUA*, *PGK* and *PPP2R3*, and *TUA* and *EF-1α*, respectively. Similar results of varied expressions in different tissues have been reported in some plant species [4,9,28]. From the above results, we also found that the expression levels of reference genes were not always stable across different tissues in *A. venetum*.

The “traditional” reference gene *TUA* has been substantiated as an equally suitable reference gene for gene expression analysis in the leaves and roots of *A. venetum*. Similar results have been reported in the leaves and roots of *Allium sativum*, *Eucalyptus globulus*, *Solanum aculeatissimum*, and *Actinidia delicious* [10,29,30,31]. Conversely, TUA was the most unstable one in *Primula forbesii* and *Prunus persicav* [32,33]. It can be seen that the screening and identification of reference genes are the prerequisite to ensure the comparison of relative gene expression levels in different tissues. 

PP2A is a trimeric enzyme consisting of three distinct subunits—the scaffold A subunit (PP2AA), the regulatory B subunit (PP2AB), and the catalytic C subunit (PP2AC). Its main function is to selectively remove phosphate groups from serine and threonine residues in proteins [27]. Recent studies have identified PP2A as a stable reference gene in *Momordica charantia* [19] and *Codonopsis pilosula* [34]. In this study, we focused on analyzing the transcriptional expression levels of three genes within the B subunit of PP2A. Our findings suggest that PPP2R3 is the most suitable reference gene in *A. venetum* stems. Therefore, we propose that genes within the B subunit of PP2A can serve as a reliable set of reference genes for future studies.

A single reference gene may lead to inaccurate quantification of target genes [10,28]. Hence, the geNorm algorithm recommends a threshold value of 0.15 to identify the minimum number of reference genes required for precise normalization across diverse tissues and different external stress conditions [8]. Figure 4 demonstrates that the V-values in leaf, stem, and root tissues of *A. venetum* were all below 0.15. Previous studies on *Allium wallichii* flower development stages [35] and kiwifruit roots [36] have shown that two reference genes effectively stabilize the relative expression levels of target genes. In our study, both single and paired reference genes were chosen to validate and analyze the expression of genes associated with proline metabolism (*P5CS*, *OAT*, *PHD*, and *GAD*). The chosen normalized reference genes exhibited higher stability in their respective tissues when assessed individually or together. Therefore, only two reference genes were sufficient for normalizing qRT-PCR data across all examined tissues in *A. venetum*. These tissue-specific stable reference genes also proved suitable for accurate normalization under PEG treatment. 

## 5. Conclusions

In this investigation, *CYP* and *TUA*, *PGK* and *PPP2R3*, and *TUA* and *EF-1α* were identified as the two most reliable reference genes in the leaf, stem, and root of *A. venetum*, respectively. Additionally, these tissue-specific stable reference genes were found to be suitable for accurate normalization under PEG treatment in *A. venetum*. *A. venetum* has a longstanding history of utilization in China for both tea and medicinal purposes. The chosen stable reference genes are poised to facilitate research on the gene function of *A. venetum* at the transcript level.

## Figures and Tables

**Table 1:** The qRT-PCR primers of reference genes and the verification genes.

Gene	Gene Name	Primer Sequences (5′-3′)	Amplicon Product Length (bp)
Abbreviation		(Forward/Reverse)	
*TUA*	*α-tubulin*	F:CTTCTTCAGCGAAACTGGGTC;	152
		R:TGTTAGCGGCGTCTTCCTTT	
*TUB*	*β-tubulin*	F:GTGGTCGTTATGTCCCTCGTG;	176
		R:ATCAATCAACTCGGCACCCT	
*ACT*	*Actin*	F:TTATCATTACCTACACCACCTCC;	135
		R:TGTAAGACTGAAGCCATCTCG	
*CYP*	*Cyclophilin*	F:AGGGTGAAAAGGAAGATGAAG;	206
		R:GTACAACAAGTTCTGGGGTGATA	
*EF-1α*	*Elongation factor-1α*	F:TTGGAGGTATTGACAAGCGAGTA;	252
		R:TCAACACAGCACAATCAGCCTG	
*PPP2R2*	*Encoding B/R2/PR55*	F:TTACATGACCCTTAAGTTATGGG;	173
		R:AGGAACCAGTTGCTACACGCA	
*PPP2R3*	*Encoding B″/R3/PR72*	F:TTTCTTATTCGTCAGGAGCGT;	129
		R:CCAAACATCTGCACTTCCGT	
*PPP2R5*	*Encoding B′/R5/PR61/B56*	F:CTTTCTTCCGTGATGTTCCAGTC;	279
		R:GGTCCACATTCTCAGACCCTGTA	
*PGK*	*3-phosphoglycerat kinase*	F:TGGCAGACAAGTTTGCTCCT;	181
		R:AAAAACTCCCATAGGTCCATT	
*P5CS*	*Δ^1^-pyrroline-5-carboxylate* *synthase*	F:CTTCATCAATCCCAGAAAGAGTT; R:ATGTCCAAGAACTGGAATTTTTGTG	173
*OAT*	*Ornithine* *δ-aminotransferase*	F:GAAGTCCACCCTGATGTCGT R:AAATGTGCTTCCATGCTCCC	126
*PHD*	*Proline hydroxylase*	F:TTTGATTATGTGCCGGAAGC R:TCTTCGGACAACCATCTCG	242
*GAD*	*Glutamic acid* *decarboxylase*	F:CAAGCAGACTGGATGGGATA R:CAATGGCAACCTAAAGTCCC	106

**Table 2:** The ranking of gene expression stability for 9 potential reference genes across various tissues of *A. venetum* using geNorm, NormFinder, BestKeeper, and RefFinder.

Treatments	geNorm		NormFinder		Bestkeeper			RefFinder		Rank
	Gene	Stability	Gene	Stability	Gene	CV	SD	Gene	Value	
Leaf	*PGK*	0.057	*PPP2R5*	0.052	*CYP*	0.04	0.17	*CYP*	1.41	1
	*CYP*	0.057	*CYP*	0.079	*PGK*	0.07	0.26	*TUA*	2.00	2
	*TUA*	0.092	*TUA*	0.080	*TUA*	0.07	0.28	*PGK*	2.51	3
	*PPP2R5*	0.161	*PGK*	0.115	*PPP2R5*	0.12	0.46	*PPP2R5*	3.00	4
	*ACT*	0.198	*ACT*	0.124	*TUB*	0.18	0.59	*ACT*	4.95	5
	*TUB*	0.213	*TUB*	0.152	*ACT*	0.19	0.70	*TUB*	5.73	6
	*PPP2R3*	0.220	*PPP2R3*	0.175	*PPP2R3*	0.19	0.70	*PPP2R3*	7.00	7
	*PPP2R2*	0.242	*PPP2R2*	0.184	*PPP2R2*	0.21	0.74	*PPP2R2*	8.00	8
	*EF-1α*	0.289	*EF-1ɑ*	0.300	*EF-1α*	0.27	1.08	*EF-1α*	9.00	9
Stem	*PGK*	0.027	*PPP2R3*	0.009	*PPP2R5*	0.00	0.02	*PPP2R3*	1.41	1
	*PPP2R3*	0.027	*PGK*	0.009	*PPP2R3*	0.03	0.10	*PGK*	1.73	2
	*PPP2R5*	0.037	*PPP2R5*	0.010	*PGK*	0.05	0.20	*PPP2R5*	2.06	3
	*CYP*	0.059	*ACT*	0.031	*CYP*	0.05	0.20	*CYP*	4.23	4
	*ACT*	0.088	*CYP*	0.048	*ACT*	0.07	0.27	*ACT*	4.73	5
	*PPP2R2*	0.124	*PPP2R2*	0.126	*PPP2R2*	0.14	0.50	*PPP2R2*	6.00	6
	*TUA*	0.159	*TUA*	0.198	*TUA*	0.19	0.76	*TUA*	7.00	7
	*EF-1α*	0.223	*EF-1α*	0.284	*TUB*	0.26	0.88	*EF-1α*	8.24	8
	*TUB*	0.271	*TUB*	0.289	*EF-1α*	0.28	1.05	*TUB*	8.74	9
Root	*EF-1α*	0.025	*CYP*	0.004	*ACT*	0.01	0.04	*ACT*	1.86	1
	*TUA*	0.025	*TUA*	0.008	*TUA*	0.02	0.06	*TUA*	2.11	2
	*ACT*	0.031	*EF-1α*	0.009	*EF-1α*	0.02	0.09	*EF-1α*	2.28	3
	*CYP*	0.047	*ACT*	0.011	*CYP*	0.04	0.17	*CYP*	2.38	4
	*PGK*	0.052	*PGK*	0.018	*PGK*	0.05	0.19	*PGK*	4.73	5
	*TUB*	0.080	*TUB*	0.102	*PPP2R5*	0.11	0.43	*TUB*	6.24	6
	*PPP2R5*	0.099	*PPP2R5*	0.103	*TUB*	0.12	0.46	*PPP2R5*	6.74	7
	*PPP2R2*	0.119	*PPP2R2*	0.121	*PPP2R2*	0.12	0.43	*PPP2R2*	8.00	8
	*PPP2R3*	0.153	*PPP2R3*	0.182	*PPP2R3*	0.17	0.59	*PPP2R3*	9.00	9

## Data Availability

The data presented in this study are available in article.

## Supplementary Materials

The following supporting information can be downloaded at: https://www.mdpi.com/article/10.3390/genes15020231/s1, Figure S1. Agarose gel electrophoresis of total RNA, Figure S2. Melting curve analysis of 9 reference genes in *A. venetum,* Figure S3. Expressional stability of candidate reference genes made by geNorm analysis, Figure S4. Expressional stability of candidate reference genes made by NormFinder analysis, Figure S5. Expressional stability of candidate reference genes made by BestKeeper analysis

## Abbreviations

qRT-PCR	Real-time quantitative polymerase chain reaction
*TUA*	α-tubulin actin
*TUB*	β-tubulin
*ACT*	Actin
*CYP*	Cyclophilin
*EF-1α*	Elongation factor-1α
*PPP2R*	B family of regulatory subunits of the protein phosphatase
*PGK*	Phosphoglycerate kinase
*P5CS*	Δ^1^-pyrroline-5-carboxylate synthase
*OAT*	Ornithine δ-aminotransferase
*PHD*	Proline hydroxylase
*GAD*	Glutamic acid decarboxylase
