# Supplementary material for: Evaluation of Suitable Reference Genes for Quantitative Real-Time PCR in Various Tissues of Apocynum venetum
Source: Genes (Basel). 2024 Feb 11;15(2):231. doi: 10.3390/genes15020231 (PMC10888412; doi:10.3390/genes15020231)
Supplement: Supplementary file 1 [file genes-15-00231-s001.zip › genes-2822319-supplementary.pdf]

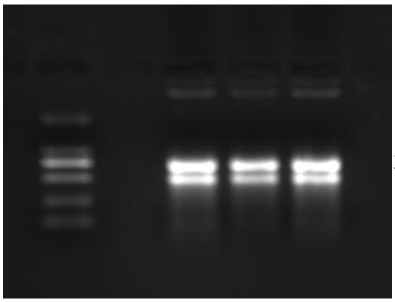

Fig. S1. Agarose gel electrophoresis of total RNA

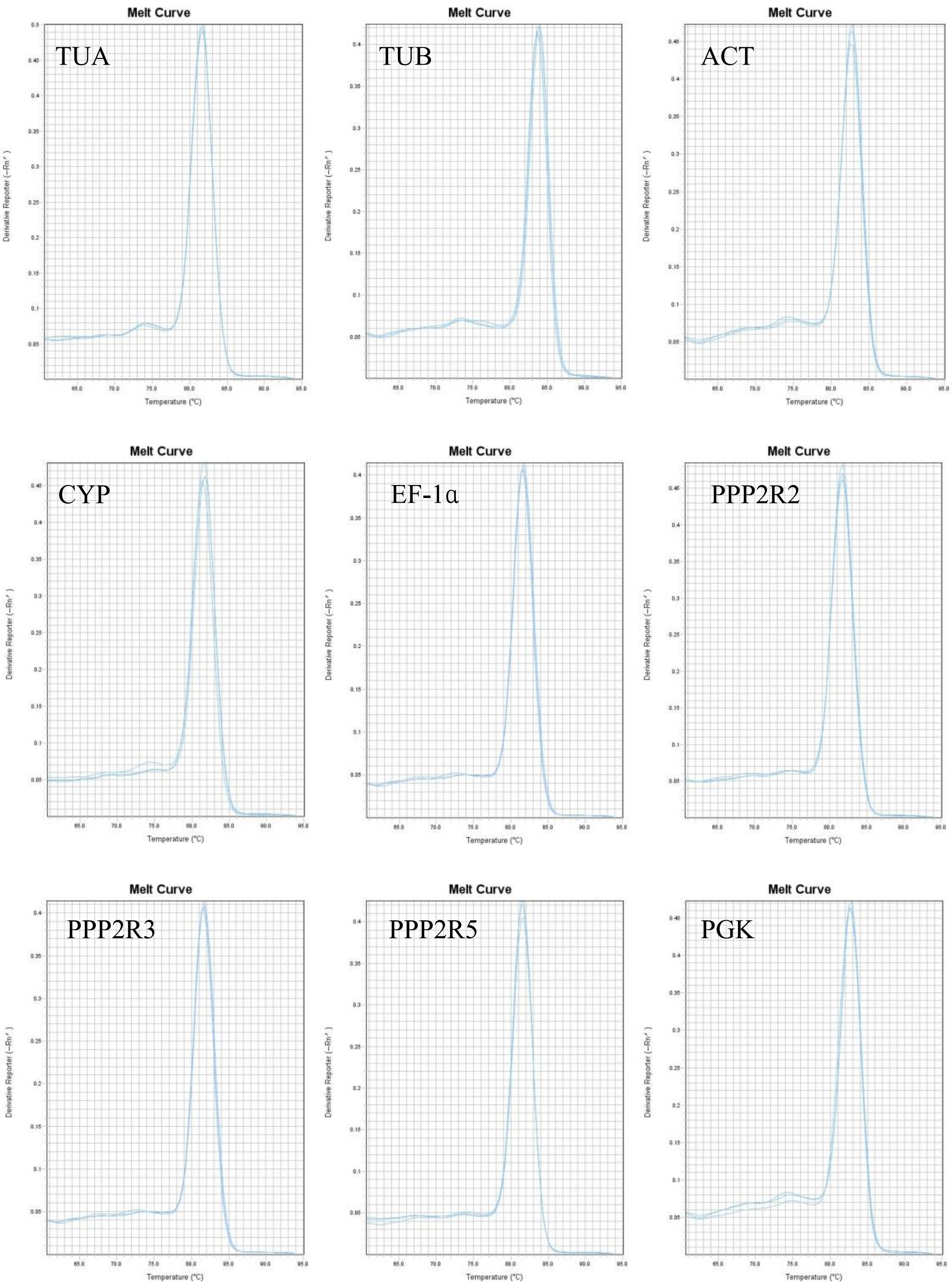

Fig. S2. Melting curve analysis of 9 reference genes in *A. venetum*

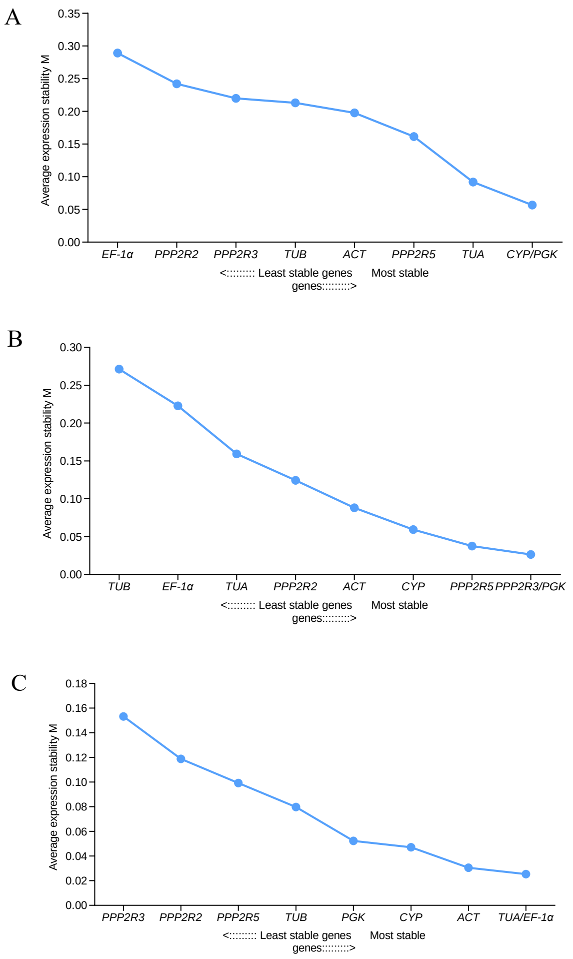

Fig. S3. Expressional stability of candidate reference genes made by geNorm analysis (A) leave (B) stems(C) root

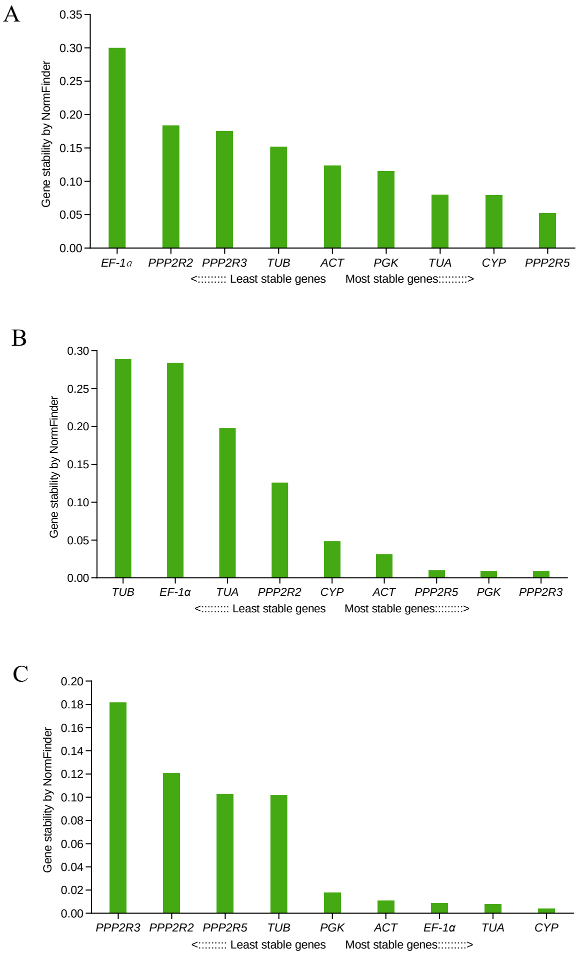

Fig. S4. Expressional stability of candidate reference genes made by NormFinder analysis (A) leave (B) stems(C) root

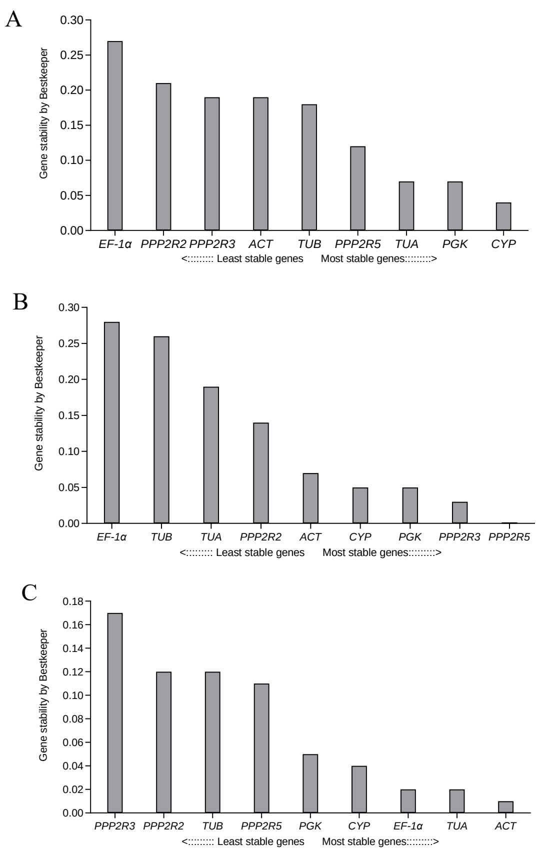

Fig. S5. Expressional stability of candidate reference genes made by BestKeeper analysis (A) leave (B) stems(C) root
